# Non-infectious mixed cryoglobulinemia as a new clinical presentation of mutation in the gene encoding coatomer subunit alpha: a case report of two adult sisters

**DOI:** 10.3389/fimmu.2024.1450048

**Published:** 2024-11-15

**Authors:** Ksymena Leśniak, Rafał Płoski, Małgorzata Rydzanicz, Aleksandra Rymarz, Arkadiusz Lubas, Tomasz Syryło, Stanisław Niemczyk

**Affiliations:** ^1^ Department of Internal Diseases, Nephrology and Dialysis, Military Institute of Medicine-National Research Institute, Warsaw, Poland; ^2^ Department of Medical Genetics, Medical University of Warsaw, Warsaw, Poland; ^3^ Department of General, Functional and Oncological Urology, Military Institute of Medicine- National Research Institute, Warsaw, Poland

**Keywords:** COPA gen, COPA syndrome, cryoglobulinemia, cryoglobulinemia vasculitis, mixed cryoglobulinemia

## Abstract

Cryoglobulinemia is a rare disease characterized by the presence of cryoglobulins in the blood serum. It is usually caused by autoimmune, lymphoproliferative, or infectious factors. The pathogenesis of cryoglobulinemia is not well understood, therefore, genetic testing is very important. We present the case of two adult sisters with different clinical phenotypes of non-infectious cryoglobulinemic vasculitis associated with a rare genetic variant [(Hg38) 1:160323529 C>G, NP_004362.2:p.(Gly203Ala)]. One of the sisters suffered from essential mixed cryoglobulinemia, while the other suffered from cryoglobulinemia associated with systemic connective tissue disease. In both cases, genetic tests revealed a variant in the COPA gene, encoding coatomer subunit alpha. Mutations in the COPA gene are associated with COPA syndrome, an autoimmune interstitial lung, joint, and kidney monogenic disease, found mainly in children. Only 15 pathogenic COPA variants have been reported thus far which suggests that the full spectrum of disease manifestations remains unknown. Ours is the first report of the association of the COPA gene with non-infectious cryoglobulinemic vasculitis in adults. This unexpected finding may direct research into the pathogenesis of cryoglobulinemia and new treatment strategies for this rare disease.

## 1 Introduction

Cryoglobulinemia is a unique disease at a crossroads between autoimmune and lymphoproliferative disorders and is characterized by the presence of cryoglobulins in the blood serum. Cryoglobulins are immunoglobulins that precipitate *in vitro* at <37°C and dissolve when heated. Once in the blood system, these molecules can cause organ damage through the occlusion of blood vessels or through vasculitis (1, 2). Cryoglobulins are produced via monoclonal or polyclonal expansion of B lymphocytes as a consequence of lymphoproliferative disorders or persistent immune stimulation caused by chronic infections or autoimmune diseases (3–5). There are three subtypes of cryoglobulins based on their clonality and the type of immunoglobulins they contain (6). Type I is closely related to hematological diseases such as multiple myeloma, Waldenstrom’s macroglobulinemia, and chronic lymphocytic leukemia (7). Types II and III [referred to as mixed cryoglobulinemia, (MC)] occur in infections [mainly Hepatitis C Virus (HCV)], autoimmune diseases [mainly systemic lupus erythematosus (SLE) and Sjögren’s syndrome (SS)] and, less frequently, cancers (type B lymphomas) (8–10). Mixed cryoglobulinemia, which is not related to the above diseases, is called “essential MC” (1).

Mixed cryoglobulinemia belongs to the group of systemic small vessel vasculitis (cryoglobulinemic vasculitis) and, due to its monoclonal component, is also a monoclonal gammopathy of clinical significance (MGCS) (11, 12). The clinical symptoms of MC range from mild (the classic triad of symptoms: purpura, arthralgia, and fatigue), through more serious neurological (peripheral neuropathy), nephrological (chronic glomerulonephritis), and hepatological manifestations (chronic hepatitis), to life-threatening systemic manifestations such as vasculitis or, less frequently, the development of cancer (11, 13–15). The proposed classification criteria for cryoglobulinemic vasculitis are based on the clinical symptoms of organ involvement and the detection of cryoglobulins in serum, but also on serological tests indicating decreased complement component 4 (C4) and increased rheumatoid factor (RF) levels (16).

Treatment of this rare disease poses a challenge and depends on the etiopathogenesis and clinical manifestations. There are three treatment strategies for cryoglobulinemia: antiviral treatment, conventional immunosuppression, and biological treatment. Rituximab, a biological drug that depletes B lymphocytes, is an essential component of modern immunosuppressive regimens in cryoglobulinemia (17, 18).

Despite years of research and observation, many aspects of cryoglobulinemia are still unclear, especially regarding the pathogenesis of this rare disease.

**Figure 1:**
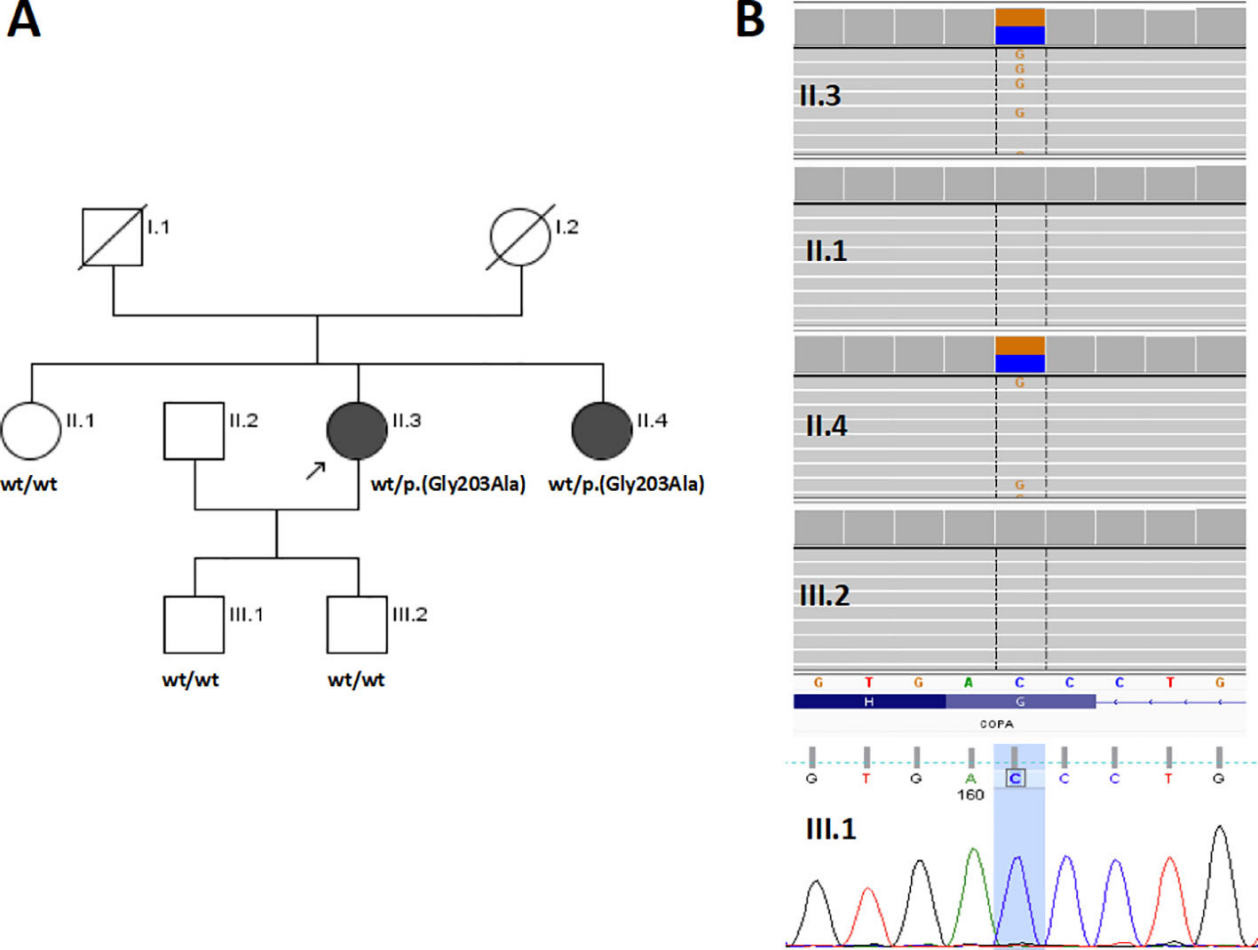
Genetic study of a Polish family with COPA syndrome. **(A)** Pedigree of the examined family with phenotype/genotype information; circles represent females, squares indicate males, filled symbols indicate an affected individual. The proband is marked with a black arrow; wt = wild-type. **(B)** The *COPA* p.(Gly203Ala) variant validation and family study results. For the amplicon deep sequencing (II.1, II.3, II.4, and III.2), an Integrative Genomic Viewer screen shot is presented. For the Sanger sequencing (III.1), a part of the chromatogram is presented.

We present the case reports of two adult sisters with non-infectious mixed cryoglobulinemia, in whom exome sequencing (ES) revealed a variant in the gene encoding coatomer subunit alpha (*COPA*) which was absent in the remaining family members, including the third healthy sister (**Figure 1**).

According to the latest reports, heterozygous mutations in the *COPA* gene (chr.1 q23.2) result in COPA syndrome, a rare autoinflammatory disease inherited in an autosomal dominant manner with incomplete penetrance and variable expression (19).

The classic COPA syndrome phenotype mainly includes interstitial lung disease, most often in the form of intra-alveolar hemorrhage, arthritis, glomerulonephritis, and high titers of circulating autoantibodies (19, 20). Most symptoms appear in early childhood, 64% by the age of 5. Thus far, only a few cases have been reported with symptoms occurring over the age of 50 years (21–23).

According to The Human Gene Mutation Database (HGMD), only 15 different high-confidence pathogenic *COPA* variants have been reported in the literature thus far, raising the possibility that the full spectrum of the disease phenotype is not known.

## 2 Cases presentation

### 2.1 Index case

**Figure 2:**
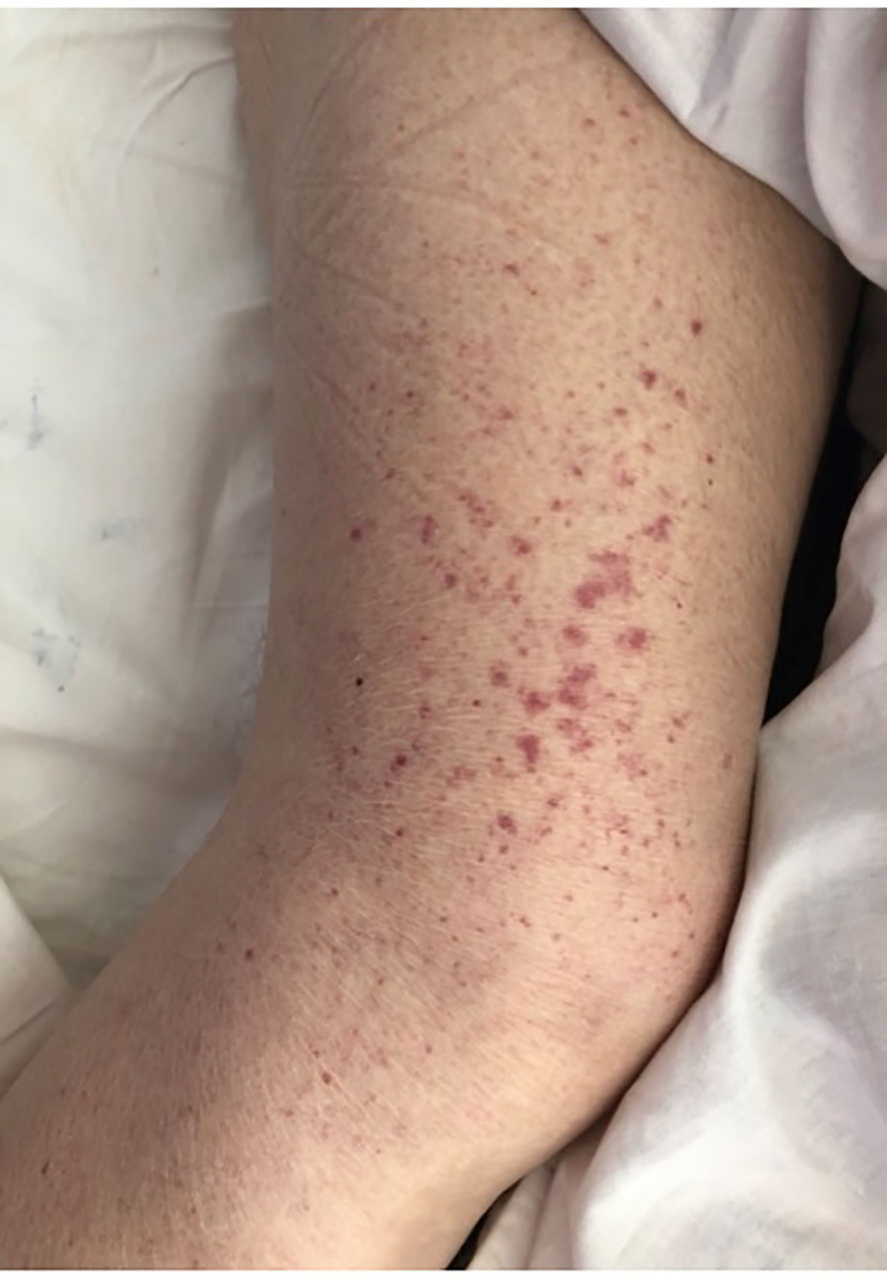
Cryoglobulinemic vasculitis presents as a small-spotted rash on the lower legs in our index case.

A 58-year-old patient with hypothyroidism in the course of Hashimoto’s disease and bipolar disorder was admitted to the Department of Nephrology in Warsaw (in 2012) for the diagnosis of purpura. A medical interview revealed pain in the upper abdomen and in the joints of the hands and lower limbs, and then a small-spotted rash on the lower legs appeared a few weeks before the admission (**Figure 2**). The patient’s family history included a sister diagnosed with cryoglobulinemia and Sjögren’s syndrome.

The physical examination revealed no abnormalities apart from purpura on the skin of the lower legs. Laboratory tests performed during hospitalization revealed increased inflammatory markers: erythrocyte sedimentation rate (ESR) of 52 mm/h, C-reactive protein (CRP) level of 3.7mg/dl; elevated rheumatoid factor (RF 64 IU/ml), and significantly reduced complement C4 (0.03 g/l). Kidney function was normal, creatinine was in the range of 0.7mg/dl, and a general urine test showed no abnormalities.

The tests for antinuclear antibodies (ANA) and antineutrophil cytoplasmic antibodies (ANCA) and those against double-stranded DNA (ds-DNA) and soluble nuclear antigens (Ro, La, Sm) were negative. Infection with hepatitis B Virus (HBV), HCV, and human immunodeficiency virus (HIV) was excluded. A chest x-ray and abdominal ultrasound showed no significant abnormalities. The abdominal pain was transient and did not recur. No changes in the gastric or duodenal mucosa were reported in gastroscopy. A skin biopsy was performed, which showed signs of leukocytoclastic small vessel vasculitis. Antinuclear antibodies were negative and the patient did not present symptoms of dryness typical of Sjögren’s syndrome, therefore the diagnostics were not further investigated. Since cryoglobulinemia was considered in the differential diagnosis, a test was performed which confirmed the presence of cryoglobulins. After immunochemical analysis which revealed the presence of polyclonal IgM and polyclonal IgG cryoglobulins, a mild form of mixed cryoglobulinemia type III was diagnosed. Immunosuppressive treatment was implemented including glucocorticosteroids (prednisone 55mg/day) and methotrexate (15mg/week). Within a few months, despite the treatment, the purpura recurred with a tendency to form confluent changes. Additionally, sensory disturbances appeared in the lower limbs, accompanied by a significant increase in the concentration of cryoglobulins (4.3 g/L). Simultaneously, a monoclonal component of the IgM class with kappa light chains appeared among the cryoglobulins. Another serological diagnosis for systemic connective tissue disease was performed but its results were negative. A bone marrow biopsy excluded multiple myeloma and other hematological malignancies. Increased inflammation markers and a decrease in complement C4 persisted. HCV RNA was unreactive. To diagnose any sensory disorders, an MRI of the head, electroneurography (ENG), and a neurological consultation were performed showing evidence of vascular brain damage (diffused, small lesions typical of cerebral vasculitis in MRI) and axonal sensory-motor polyneuropathy, most likely in the course of cryoglobulinemic vasculitis. The patient was diagnosed with essential mixed cryoglobulinemia type II. Due to the lack of effectiveness of the current treatment and the signs of disease progression with the involvement of the nervous system, it was decided to intensify the treatment and change the therapy to monthly intravenous cyclophosphamide (CYC) infusions. During the treatment, despite the administration of 5 g of CYC, no response with a decrease in cryoglobulins was obtained and the purpura recurred. Moreover, abdominal pain, nausea, musculoskeletal pain, very severe pain in the lower limbs with a burning sensation and numbness in the feet, increasing swelling of the lower legs, bruising of the three toes of the left foot, and a low-grade fever occurred. Cryoglobulins were still present in the blood serum. Due to the risk of necrosis of the toes, the patient qualified for therapeutic plasma exchange (TPE) procedures. A total of 5 TPE procedures were performed without complications and then another CYC pulse therapy was administered. The treatment resulted in the improvement of both the general condition and the blood supply to the left toes, however, disease remission was not achieved. Due to the lack of effectiveness of the current therapy, it was possible to use rituximab in the Polish conditions. It was decided to administer rituximab (RTX, 1.0 g on days 1 and 15) and prednisone was continued with a gradual reduction of the dose. A transient clinical remission of the disease was achieved without cryoglobulin negativity. The lymphocyte CD19 population was regularly determined by flow cytometry, but these results did not significantly influence therapeutic decisions.

One year after RTX administration, inflammatory state parameters and the cryoglobulin concentration increased and symptoms of kidney involvement appeared in the form of hematuria. Therefore, it was decided to start treatment with mycophenolate mofetil (MMF) and prednisone administration was continued. The clinical remission with cryoglobulin negativity was achieved but lasted only 8 months. The disease then relapsed, requiring the administration of a second course of rituximab. Prednisone was continued at a low dose, and after a few months, MMF treatment was added which resulted in a year-long remission of the disease with negative cryoglobulins.

Due to the high risk of recurrence of cryoglobulinemia symptoms and associated complications and the confirmed high effectiveness and safety of the previously used biological treatment, the patient received booster doses of rituximab (after 4 and 6 years of the disease). Despite complex immunosuppressive therapy, the patient’s cryoglobulinemic vasculitis could not be sufficiently controlled. The disease kept recurring and in its final stage, 8 years after diagnosis, an ischemic stroke occurred. Repeated emergency TPE procedures were planned, but they were not carried out. The patient died in 2019 as a result of COVID-19-related severe respiratory failure (before the availability of vaccinations).

### 2.2 Sister

A 50-year-old patient with a history of Sjögren’s syndrome and Hashimoto’s disease for approximately 10 years was admitted to the Rheumatology Department in Cracow (2007) due to recurrent purpura on the lower limbs for 2 years with accompanying pain in the limbs. Physical examination revealed abnormalities including purpura on the skin of the lower legs and thighs, which was confluent in some places, discoloration in sites where the changes disappeared, sensory disturbances in the distal parts of the lower limbs, and persistent dryness of the eyes and mouth. Moreover, abnormal test results included leukopenia in the blood count, accelerated ESR (50mm/h) with normal CRP, and a decrease in both complement components, especially C4 (C3: 0.768 g/L; C4: 0.019 g/L). Serological tests revealed the presence of rheumatoid factor with a negative anti-cyclic citrullinated peptide antibodies (anti-CCP) result and the presence of antinuclear antibodies with a homogeneous + granular type (titer of:20408) and mitochondrial type (titer of:5120). Tests for dsDNA, nRNP, Sm, SS-A (Ro), SS-B (La), Scl-70, PM-Scl, Jo-1 and ANCA were negative. However, cryoglobulins were detected in blood serum. Hepatitis B, hepatitis C, and HIV infections were excluded. TSH level was normal. A chest x-ray revealed fibrosis in the lower parts of the lungs. The abdominal ultrasound examination did not show any abnormalities. Cryoglobulinemia with the involvement of the skin and peripheral nervous system secondary to systemic connective tissue disease (CTD) was diagnosed and treatment with methylprednisolone was initiated at the initial dose of 16mg/day which resulted in clinical remission. After a year, anti-Ro-52 (+++) antibodies were detected and after 13 years anti-SCL 70 (+) antibodies appeared. Hydroxychloroquine 250mg/day was added to the glucocorticoids. Due to the history of cryoglobulinemia and persistent C4 hypocomplementemia, the patient was subjected to strict surveillance, considering the higher risk of developing lymphoma.

**Figure 3:**
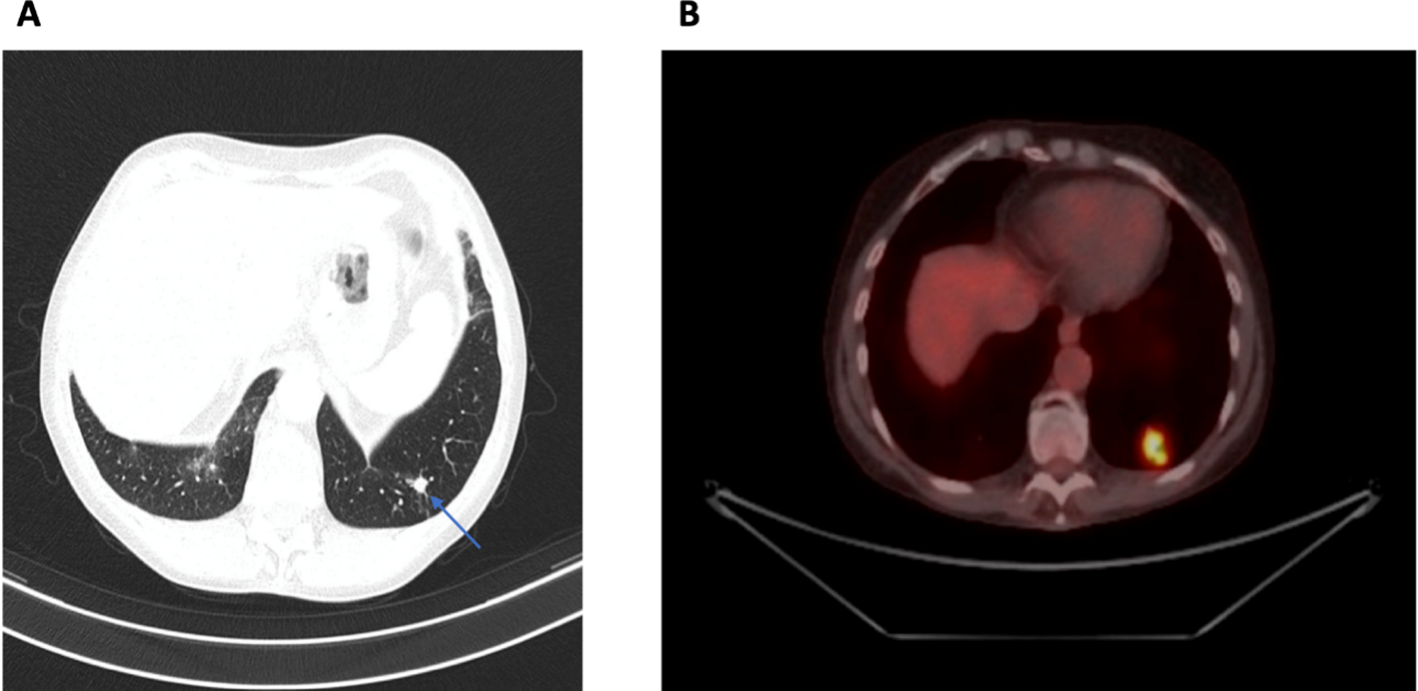
**(A)** A CT scan of the chest revealed a left lung tumor (blue arrow). **(B)** FDG-PET/CT examination revealed a polycyclic nodular lesion of the left lung with dimensions of approx. 25x17mm, with a maximum standardized uptake value (SUVmax) of 11.6.

After 11 years, in the absence of any respiratory symptoms, a follow-up CT scan of the chest revealed a left lung tumor (**Figure 3A**). The positron emission tomography-computed tomography (PET-CT) [on 18F-fluorodeoxyglucose-positron emission tomography (FDG-PET/CT)] revealed a polycyclic nodular lesion of the left lung with dimensions of approx. 25x17mm, with increased glucose metabolism (**Figure 3B**). It was decided to remove segment 10 of the left lung. Histopathological examinations of sections from this lesion demonstrated granulomas composed of epithelioid and giant cells accompanied by an abundant inflammatory component of lymphoid cells, foci of necrosis in some areas, fibrosis of the perivascular parenchyma, and areas of destruction of the bronchiolar wall. First, tuberculosis and other bacterial and fungal infections were ruled out. Due to the lack of typical clinical features, sarcoidosis was also ruled out. The test for ANCA was negative and cryoglobulins were still detected in the blood serum. Based on subsequent chest CT scans (which found ground glass zones, subpleural densities, small nodules, and small fibrosis in the lungs), interstitial lung disease closely resembling nonspecific interstitial pneumonia (NSIP) in the course of Sjögren’s syndrome was diagnosed and therefore, oral MMF was added to previous glucocorticoids in the treatment. The treatment provided stabilization which was visible in lung imaging tests, however, the patient developed symptoms of bronchial asthma. In the pulmonary function testing (PFT), an obstructive ventilatory defect was found and the patient required inhalant drugs.

Finally, 17 years after the diagnosis of cryoglobulinemia, the symptoms of the disease have not recurred, the patient feels well, and she is under the care of a rheumatologist and pneumonologist.

### 2.3 Genetic study - methods

Genomic DNA was extracted from the whole blood of all study participants using standard methods. In the index case (proband), MONO ES was performed using a Twist Human Core Exome 2.0 + Comp Spike-in + Twist mtDNA Panel (Twist Bioscience, South San Francisco, CA, USA). The enriched library was pair-end sequenced (2x100 bp) on a NovaSeq 6000 (Illumina, San Diego, CA, USA) to a mean coverage of 120x, GE10 (coverage greater or equal 10) = 98%, GE20 = 97.7%. The raw data analysis and variants prioritization were performed as previously described (24). *In silico* pathogenicity prediction was performed based on the in-house developed platform GeneBe (https://zgm.wum.edu.pl/cloud2/) and the American College of Medical Genetics and Genomics (ACMG) guidelines (25, 26). Conservation scores were provided by Varsome (27).

Segregation of the variant considered causative was further examined in all available members of the proband’s family, including two sisters (unaffected II.1 and affected II.4) and two of the proband’s unaffected sons (III.1 and III.2) (**Figure 1**). Variant validation and family study were performed by amplicon deep sequencing in II.1, II.3, II.4, and III.2 using a Nextera XT Kit (Illumina) sequenced as described above for ES, and for III.1 by direct Sanger sequencing with a BigDye Terminator v3.1 Kit (Applied Biosystems, Foster City, USA) on an ABI 3500Xl Genetic Analyzer (Applied Biosystems).

The study protocol was approved by the local ethics committee (Military Institute of Medicine Bioethics Committee: approval number 37/WIM/2018). Written informed consent was obtained from all participants or their legal guardians in accordance with the Declaration of Helsinki.

### 2.4 Genetic study – results

In the index case, a novel [(Hg38) 1:160323529 C>G, NM_004371.4:c.608G>C, NP_004362.2:p.(Gly203Ala)] heterozygous missense variant located in exon 8 of the *COPA* gene was identified and prioritized as disease-causing. The p.(Gly203Ala) has 0 frequency in the gnomAD v4.0.0 database (accessed 11 March 2024) and the in-house database of Polish individuals composed of >11,000 exomes (accessed 11 March 2024). The variant was predicted to be pathogenic by AlphaMissense (0.95), CADD (32), Eigen_PC (0,73), FATHMM_MKL (0,99), IST_S2 (1,0), PrimateAI (0,91), and Polyphen (0,99); according to an automatically implemented ACMG classification, the variant was scored as VUS (Variant of Uncertain Significance, 4 points, PM2 Moderate, PP2 Supporting, PP3 Supporting) (25). However, when the location in a mutation hot spot region was taken into account (PM1 moderate, see Discussion) the classification changed to Likely Pathogenic (6 points). The c.608G variant is evolutionarily conserved (phyloP100 score: 7.281) and is located in a region moderately constrained for coding variation (CCRS=82.2). The family study revealed the presence of p.(Gly203Ala) in the index case’s affected sister (II.4) and its absence in index case’s unaffected sister (II.1) and both unaffected sons (III.1, III.2) (**Figures 1A, B**).

## 3 Discussion

COPA syndrome, also known as an autoimmune interstitial lung, joint, and kidney disease (MIM # 616414), is a rare autosomal dominant disorder caused by heterozygous missense variants located in exons 8 and 9 of the *COPA* gene and highly conserved WD40 domain of the COPA protein (28, 29). However, incomplete penetrance with unaffected carriers is also reported (19, 30).

In our study, we present a novel *COPA* p.(Gly203Ala) missense variant in a Polish family with two affected sisters presenting with symptoms of cryoglobulinemic vasculitis. While the pathogenicity of the variant cannot be proven, it has a very low population frequency (absent from available database) and is predicted to be so by a number of *in silico* tools including the powerful recently-developed AI-based AlphaMissense (31). Furthermore, all pathogenic *COPA* variants reported thus far in the literature cluster in exons 8 and 9 between amino acids 230-285 (19, 21, 23, 32–42) with only a single exception for position 75 (41). This clustering is also apparent when ClinVar-reported pathogenic/likely pathogenic *COPA* variants are analyzed (6 variants reported in amino acid regions 230-245, visualized at https://gnomad.broadinstitute.org/gene/COPA accessed 10 05 2024). The location of the variant we report in the proximity of the abovementioned domain (exon 8, amino acid pos. 203) is another argument supporting its pathogenicity, especially when considering that the *COPA* gene consists of 33 exons, encoding 1225 aa. It is noteworthy that after inclusion of this information, the ACMG classification of *COPA* p.(Gly203Ala) changed to Likely Pathogenic.

The presented cases are of particular importance because they concern a rare form of non-infectious cryoglobulinemia that could not be explained by known causes.

### 3.1 Genetic studies in cryoglobulinemia

In recent years, various studies of the pathogenesis of cryoglobulinemia have been conducted, but they mostly focused on patients with HCV infection. For example, *Zignego* et al. provided the results of a multicenter genome-wide association study (GWAS) focusing on the analysis of HCV-related cryoglobulinemia at the genome level which revealed a significant association between cryoglobulinemic vasculitis and the presence of single nucleotide polymorphisms (SNPs) in the vicinity of the *NOTCH4* and *MHC class II* genes on chromosome 6 (43). In turn, *Gragnani* et al. demonstrated a linkage between two polymorphisms that was previously confirmed in the HCV-related MC (NOTCH4 rs2071286 and HLA-II rs9461776) in patients with HCV-related lymphoproliferative disorders (LPDs) at risk of non-Hodgkin’s lymphoma (NHL) (44).

Therefore, it was surprising that the index case patient and her affected sister were carriers of a mutation typical for Mendelian syndrome of immune dysregulation.

### 3.2 COPA syndrome: genotype

In recent decades, many important discoveries have been made in the field of genetically determined diseases related to the dysregulation of the immune system. Thus far, over 400 different phenotypes of single-gene inborn errors of immunity (IEIs) have been described, which include not only infections but also allergies, autoimmunity, autoinflammation, and malignancy.

One of these is coatomer subunit alpha (COPA) syndrome, classified by the International Union of Immunologic Societies (IUIS) expert committee on inborn errors of immunity under the “auto-inflammatory, other” category (45). COPA syndrome can be defined as a complex disorder of the immune system, both at the innate (autoinflammatory) and adaptive (autoimmune) levels (19, 38, 46).*COPA* codes for the coatomer subunit alpha protein, a part of the coat protein complex I (COPI), which, as a component of the cell’s vesicular transport mechanism, is responsible for the retrograde movement of proteins from the Golgi apparatus back to the endoplasmic reticulum (ER) (19). The presence of a COPA mutation is associated with ER stress, which results in the release of pro-inflammatory cytokines and an expansion of T-helper type 17 (TH17) cells (19). In turn, alterations in T lymphocyte populations promote the dysregulation of the immune system and favor autoimmune diseases (47). Recent research indicates a key role of the stimulator of interferon genes (STING), a master regulator of innate immunity, in the pathogenesis of COPA syndrome (38). In patients with the *COPA* mutation, the accumulation of activated STING in the cell results in increased type I interferon (IFN) signaling (21, 48). The production of IFN type I is increased, similar to another well-described type I interferonopathy STING-associated vasculopathy with onset in infancy (SAVI) (49).

The finding of a mutation in the *COPA* gene in sisters with cryoglobulinemia, which is a lymphoproliferative disorder, may therefore indicate a relationship between intracellular disorders and the inappropriate activation of B cells, which requires further research.

### 3.3 COPA syndrome: phenotype

It appears that *COPA* gene mutations do not show a genotype-phenotype correlation for the typical symptoms of COPA syndrome, although the strength of any conclusive statement is limited due to the recent identification of this disease and a limited number of patients. COPA syndrome is an ultra-rare disease with a limited number of cases reported thus far. It is diagnosed mainly in children, hence it is rarely included in a differential diagnosis in the adult population (50). The most characteristic symptom of COPA syndrome is lung involvement; however, the symptoms of the disease may change over time in individual patients along with the appearance of autoantibodies and affect other organs and systems, sometimes leading to their failure and the need for transplantation. Pulmonary involvement in young children is often manifested by intra-alveolar bleeding, while later, interstitial lung disease (ILD) involving cysts/nonspecific interstitial pneumonia (NSIP) (on imaging) and follicular bronchiolitis, lymphocytic interstitial pneumonia, and/or interstitial fibrosis (on lung biopsy) can be observed (19, 21, 51, 52). More than 90% of patients with COPA syndrome have joint pain or arthritis, and approximately 40% of them, mainly in the second decade of life, show symptoms of kidney involvement (glomerulonephritis on biopsy). Laboratory tests reveal increased inflammatory state parameters and the presence of autoantibodies in the blood serum (ANA, ANCA, and/or RF/CCP) (19, 20, 28). Although the term “COPA syndrome” is typically reserved for cases with pulmonary involvement with or without joint or renal involvement, the COPA syndrome phenotype is widening (23). Moreover, in patients with COPA syndrome, especially in later childhood or adolescence, symptoms such as intra-alveolar hemorrhage or glomerulonephritis suggest SLE or ANCA-vasculitis, which may pose diagnostic difficulties (22, 53). The sisters with a mutation in the *COPA* gene in this case report did not present with “typical COPA syndrome” but it is well-known that a monogenic disease may have different phenotypes (54, 55). First of all, our patients were adults, but it should be emphasized that the diagnosis of a rare disease in both suggested a genetic basis. There are a few reports in the available literature that describe sisters suffering from cryoglobulinemia and brothers suffering from cryoglobulinemia and macroglobulinemia, however, the data comes from a time when genetic tests were not accessible (56–58).

Some typical features of the COPA syndrome, including interstitial lung disease and positive autoimmune serologies, were described in the sister of our index case (**Table 1**). However, a histopathological examination of the tumor in the left lung revealed granulomas, which have not been previously reported in COPA syndrome (19, 37, 52). In contrast to ANCA-positive vasculitis, granulomas are not a typical manifestation of lung involvement in cryoglobulinemia (1). In turn, the clinical picture of the index case was dominated by symptoms of cryoglobulinemic small vessel vasculitis, mainly involving the skin and the peripheral/central nervous system, which are organs less frequently affected in COPA syndrome (**Table 1**) (19, 34). Symptoms of vasculitis in the course of cryoglobulinemia were reported in both sisters. According to current knowledge, cryoglobulinemic vasculitis can also cause pulmonary alveolar hemorrhage and glomerulonephritis, which are a typical presentation of COPA syndrome (14, 59). The assessment of cryoglobulins is included in the differential diagnosis of renal-pulmonary syndrome in adults so this may be why we have not found any information in the literature about cryoglobulins in COPA syndrome predominantly diagnosed in young children.

**Table 1:** Clinical and serological characteristics of the index case and her sister with cryoglobulinemia.

	Index case	Sister
Sex	F	F
Age of onset	58 years	50 years
Skin	Purpura; Toe ischemia	Purpura
Joints	Joints pain	–
CNS/Peripheral nerve	Sensory-motor polyneuropathy Vascular changes of the CNS Ischemic stroke	Sensory-motor polyneuropathy
Pulmonary manifestation	–	Lung granulomas Interstitial lung disease Asthma
DAH	–	–
Renal manifestation	Hematuria	–
Cryoglobulins	Positive	Positive
C3	Normal	↓
C4	↓↓	↓↓
RF	↑	↑
ESR	↑	↑
HCV	Neg.	Neg.
HBV	Neg.	Neg.
HIV	Neg.	Neg.
ANA	Neg.	Positive (anti Ro-52, anti SCL-70)
dsDNA	Neg.	Neg.
ANCA	Neg.	Neg.
Anti CCP	Neg.	Neg.
*COPA* mutation	p. (Gly203Ala)	p. (Gly203Ala)
Co-morbidity	Hashimoto	Hashimoto Sjögren
Therapy	Steroids MTX CYC MMF TPE RTX	Steroids MMF

DAH, diffuse alveolar hemorrhage; CYC, cyclophosphamide; MTX, methotrexate; MMF, mycophenolate mofetil; TPE, therapeutic plasma exchange; RTX, rituximab. ↓, decreased; ↑, increased.

Vasculitis may be the leading symptom or accompanying feature in many monogenic autoinflammatory diseases (60–66). Furthermore, COPA syndrome, along with other autoinflammatory diseases such as SAVI, CANDLE (chronic atypical neutrophilic dermatosis with lipodystrophy and elevated temperature), and Aicardi–Goutières syndrome can be classified as monogenic diseases with vasculitis (60, 61). If we examine the histopathological results of the lungs of patients with COPA syndrome, we can find signs of vasculitis in most patients with necrosis of the capillary walls, and neutrophils are often identified along the capillaries, which could suggest an immunological origin of pulmonary hemorrhages (20).

### 3.4 Impact of treatment

Currently, there is no specific drug for COPA syndrome, and its symptoms are treated with immunosuppression regimens used in immunological disorders since these features dominate the clinical picture of COPA syndrome. Based on recent reports demonstrating an increased expression of type 1 interferon (IFN)-stimulated genes (ISGs) in patients with a mutation in the *COPA* gene, it has been assumed that drugs blocking type I interferon signaling (i.e., Janus kinase inhibitors such as baricitinib and ruxolitinib) may be effective (48). Indeed, these drugs have been used in patients with autoinflammatory interferonopathies including COPA syndrome (67–69).

Thus far, reports show short-term results, and greater clinical experience is required for definitive recommendations. Considering the multidrug resistance in the course of cryoglobulinemia in our index case patient, we should perhaps use interferon pathway inhibitors as the next line of treatment, however, genetic tests were performed at the end of the patient’s life, precluding a possible trial.

## 4 Conclusions

The finding of a mutation in the *COPA* gene in sisters with non-infectious mixed cryoglobulinemia is a valuable contribution to research on the pathogenesis of this rare disease. Moreover, the results of the genetic tests of the presented cases allow us to expand the phenotype of COPA syndrome in adults to include cryoglobulinemic vasculitis.

## Data Availability

The raw data supporting the conclusions of this article will be made available by the authors, without undue reservation.
